# Electron and Positron Scattering Cross Sections from CO_2_: A Comparative Study over a Broad Energy Range (0.1–5000 eV)

**DOI:** 10.1021/acs.jpca.2c05005

**Published:** 2022-09-07

**Authors:** Ana I. Lozano, Adrián García-Abenza, Francisco Blanco Ramos, Mahmudul Hasan, Daniel S. Slaughter, Thorsten Weber, Robert P. McEachran, Ronald D. White, Michael J. Brunger, Paulo Limão-Vieira, Gustavo García Gómez-Tejedor

**Affiliations:** † Instituto de Física Fundamental, Consejo Superior de Investigaciones Científicas, Serrano 113-bis, 28006 Madrid, Spain; ‡ Laboratório de Colisões Atómicas e Moleculares, CEFITEC, Departamento de Física, Faculdade de Ciências e Tecnologia, Universidade NOVA de Lisboa, 2829-516 Caparica, Portugal; § Departamento de Estructura de la Materia, Física Térmica y Electrónica e IPARCOS, Universidad Complutense de Madrid, Avenida Complutense, E-28040 Madrid, Spain; ∥ Chemical Sciences Division, Lawrence Berkeley National Laboratory, Berkeley, California 94720, United States; ⊥ Department of Physics and Astronomy, University of Nebraska−Lincoln, Lincoln, Nebraska 68588, United States; # The Research School of Physics, Australian National University, Canberra, ACT 0200, Australia; ¶ College of Science and Engineering, James Cook University, Townsville 4810, Australia; ○ College of Science and Engineering, Flinders University, GPO Box 2100, Adelaide, SA 5001, Australia; ∇ Department of Actuarial Science and Applied Statistics, Faculty of Business and Management, UCSI University, Kuala Lumpur 56000, Malaysia

## Abstract

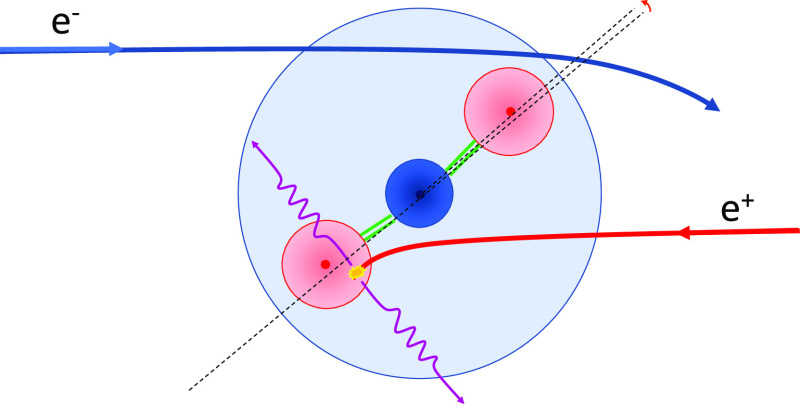

In this Review, we present a comparative study between electron and positron scattering cross sections from CO_2_ molecules over a broad impact energy range (0.1–5000 eV). For electron scattering, new total electron scattering cross sections (e-TCS) have been measured with a high resolution magnetically confined electron beam transmission system from 1 to 200 eV. Dissociative electron attachment processes for electron energies from 3 to 52 eV have been analyzed by measuring the relative O^–^ anion production yield. In addition, elastic, inelastic, and total scattering cross section calculations have been carried out in the framework of the Independent Atom Model by using the Screening Corrected Additive Rule, including interference effects (IAM-SCARI). Based on the previous cross section compilation from Itikawa (J. Phys. Chem. Ref. Data, 2002, 31, 749−767) and the present measurements and calculations, an updated recommended e-TCS data set has been used as reference values to obtain a self-consistent integral cross section data set for the elastic and inelastic (vibrational excitation, electronic excitation, and ionization) scattering channels. A similar calculation has been carried out for positrons, which shows important differences between the electron scattering behavior: e.g., more relevance of the target polarization at the lower energies, more efficient excitation of the target at intermediate energies, but a lower total scattering cross section for increasing energies, even at 5000 eV. This result does not agree with the charge independence of the scattering cross section predicted by the first Born approximation (FBA). However, we have shown that the inelastic channels follow the FBA’s predictions for energies above 500 eV while the elastic part, due to the different signs of the scattering potential constituent terms, remains lower for positrons even at the maximum impact energy considered here (5000 eV). As in the case of electrons, a self-consistent set of integral positron scattering cross sections, including elastic and inelastic (vibrational excitation, electronic excitation, positronium formation, and ionization) channels is provided. Again, to derive these data, positron scattering total cross sections based on a previous compilation from Brunger et al. (J. Phys. Chem. Ref. Data, 2017, 46, 023102) and the present calculation have been used as reference values. Data for the main inelastic channels, i.e. direct ionization and positronium formation, derived with this procedure, show excellent agreement with the experimental results available in the literature. Inconsistencies found between different model potential calculations, both for the elastic and inelastic collision processes, suggest that new calculations using more sophisticated methods are required.

## I Introduction

Electron and positron collision processes play an important role both in fundamental particle scattering studies and technological applications. In addition, since they constitute an accessible elementary particle–antiparticle pair, comparison between their respective scattering properties from atomic and molecular targets has been the subject of numerous studies in order to check model potential approximations or simply to look for more general symmetry laws. Experimental studies using the same scattering conditions for electrons and positrons have been carried out by different groups through the past decades. A comprehensive compilation of these studies was published by Kauppila and Stein^1^ in 1989. Later on Kimura el al.^2^ extended the comparison by including new experimental data and discussing some related theoretical aspects. Further experimental comparisons on electron and positron scattering by different carbon containing molecules were published by Kimura, Sueoka and collaborators.^3−6^ In 2017, Brunger et al.^7^ compiled experimental positron scattering cross sections from molecules, including total and vibrational excitation cross sections for CO_2_, for transport studies and benchmarking theory. On the other hand, model potential calculations of positron scattering by atoms have been carried out to study the role of the static and polarization potentials in elastic scattering^8,9^ and, including an absorption potential, they also provided total scattering cross section values.^10^ These model potential methods are, in principle, accurate only for intermediate and high energies (0.1–10 keV) and do not distinguish between different inelastic channels (excitation, positronium formation, and ionization) which are summed.^11,12^ For the lower energies, “ab initio” methods (R-matrix,^13^ Schwinger Multichannel,^14,15^ Convergent Close Coupling^16^), traditionally used for electron scattering, were also applied to the case of positrons. Although these methods produced accurate results for elastic scattering, they have difficulties incorporating the important inelastic channels, such as positronium formation. Specific inelastic scattering processes, including positron-atom bound state and positronium formation, have been also calculated (see for instance previous studies from Bartschat,^17^ Mitroy and Ratnavelu,^18^ or Dzuba et al.^19^). For the aforementioned theoretical methods, positron scattering calculations have been extended to molecular targets (see, for instance, publications from Tennyson,^20^ Blanco et al.,^21^ Joshipura et al.,^22^ da Silva et al.,^23^ and Zamit et al.^24^). A recent summary on the state of positron scattering from atomic and molecular databases has been published by Nahar and Anthony.^25^ For the inelastic part of the scattering, additional discussions involving difficulties on modeling positronium formation^26^ and inconsistencies between experiments and calculations, with respect to electronic excitation, have been published.^27^ In spite of the large number of theoretical and experimental studies devoted to this topic no general consensus has yet been found about the trend of the main electron and positron scattering processes from molecules, as a function of the impact energy, especially for the lower and higher energies considered in those studies. These considerations motivated the present experimental and theoretical study, in which experimental total scattering cross sections for electron and positron collisions with CO_2_ are revisited. Accurate new total electron scattering measurements have been carried out in order to obtain reference data for a cross section comparative analysis, which we have then performed for different scattering processes (elastic, ionization, electronic, and vibrational excitation). Total electron and positron scattering cross sections have then been calculated using our Independent Atom Model with the Screening Corrected Additivity Rule,^28^ including Interference effects,^29^ the IAM-SCARI method. A detailed comparison between these electron and positron calculated cross sections will provide relevant conclusions about the general energy dependence of the TCS, and the contribution of specific scattering channels (elastic, excitation, positronium formation, and ionization) for each projectile over a broad energy range (0.1–5000 eV). The remainder of this paper is organized as follows: new total electron scattering cross section measurements are presented in Section II (experimental method, results, and comparison with previous data). Electron and positron elastic scattering calculations using our IAM-SCARI method are presented and discussed in Section III. In Section IV, a comparative study on electron and positron scattering data is carried out at the level of the integral elastic and the different inelastic (electronic excitation, positronium formation and ionization) cross section levels, with some recommended data being compiled in Section V. Finally, some conclusions are drawn in Section VI.

## II Electron Scattering Cross Section Measurements

Electron scattering cross sections from CO_2_ have been the subject of numerous theoretical and experimental studies. A comprehensive review of the main results of these studies was published by Itikawa^30^ in 2002, including recommended electron scattering cross section values for the different scattering processes. Apart from checking the accuracy of the cross sections recommended in that review and updating these data through a critical evaluation of available information, an additional motivation for us is to provide new total cross section (TCS) measurements for incident electron energies ranging from 1 to 200 eV. From the analysis of the observed local maxima in the experimental TCS values, electron scattering resonances can be identified. These resonances correspond to electron attachment processes, many of them leading to molecular dissociations (Dissociative Electron Attachment), which are very important to properly model electron transport in gases. Many experimental and theoretical studies have been devoted to describe resonant electron scattering by CO_2_ molecules.^31−39^ In particular, special attention has been paid to the theoretical determination of the position and structure of the ^2^Π_u_ resonance and the existence of a virtual state near zero energy.^40−42^ However, probably due to energy resolution limitations, most of those resonances are not appreciable in the TCS values recommended by Iitakawa.^30^ In fact the only well-defined feature resolvable in his recommended data is the prominent peak around 3.8 eV, which has been assigned to the mentioned ^2^Π_u_ shape resonance.^33^ Related studies involving vibrational excitation of CO_2_ by electron impact can also be found in the literature.^40−51^

### II.A Total Electron Scattering Cross Section Measurements

**Figure 1:**
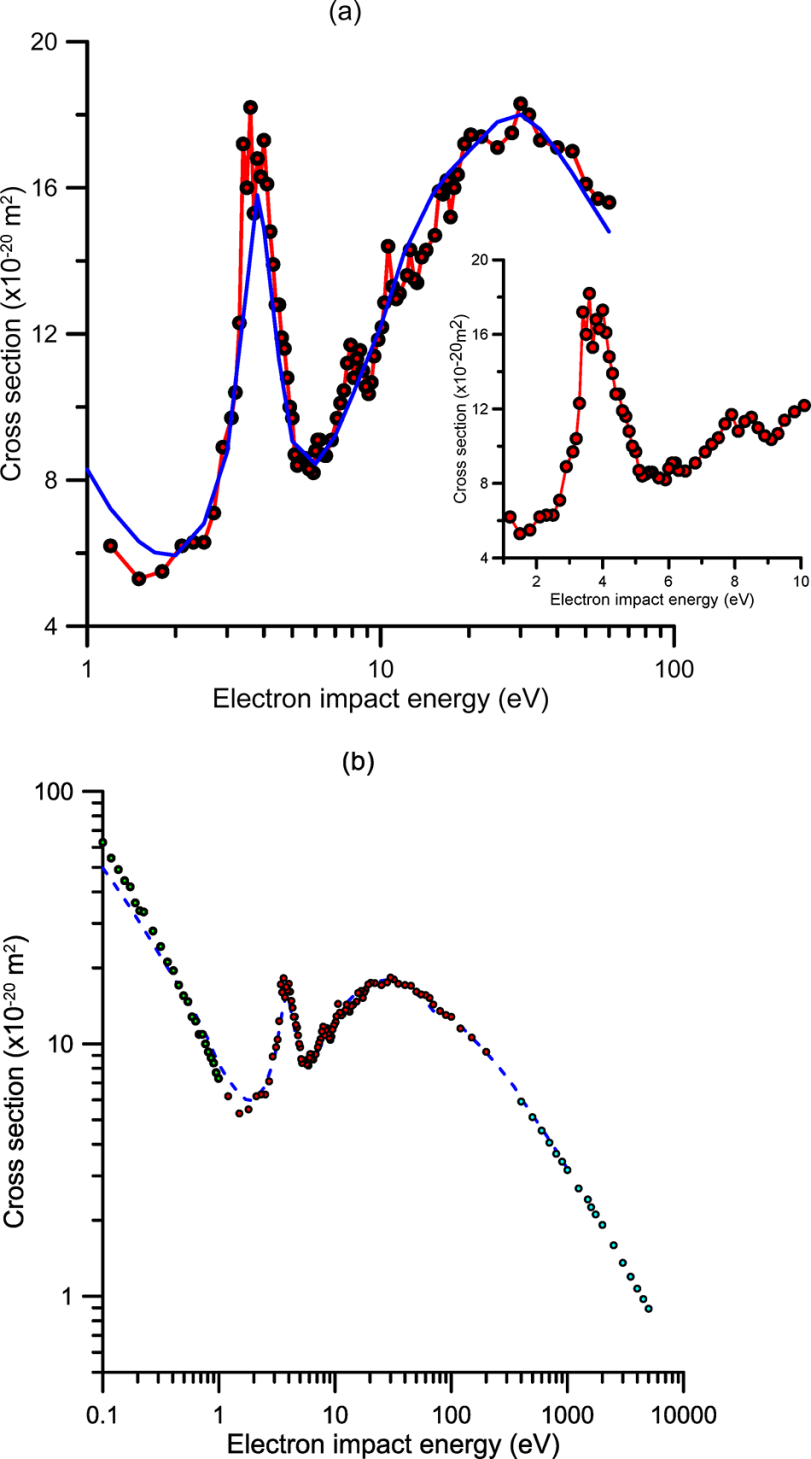
Total electron scattering cross sections (TCS) from CO_2_. (a) Key: red ●, present experimental data; blue −, recommended data from ref (30). Inset, detail of the present experimental TCS for impact energies below 10 eV. (b) Key: red ●, present experimental data; green ●, low energy results from ref (55); high energy data from ref (54); blue ---, recommended values from ref (30).

The present TCS measurements have been carried out with our magnetically confined electron-beam-transmission apparatus,^52^ which has been recently modified^53^ in order to improve the energy resolution (currently about 80 meV). Details on the experimental setup and measurement protocols can be found in ref (52). The corresponding experimental TCS results, with total uncertainty limits within ±5%, in the impact energy range 1–200 eV, are shown in Table 1 and plotted in Figure 1a. This figure contains an inset, for impact energies below 10 eV, showing the structures discussed in the next subsection.

**Table 1:** Present Total Electron Scattering Cross Section (TCS) as Measured with a Magnetically Confined Electron Transmission Apparatus (See Text for Details)

energy (eV)	TCS (10^–20^ m^2^) uncertainty (±5%)	energy (eV)	TCS (10^–20^ m^2^) uncertainty (±5%)
1.2	6.2	8.5	11.6
1.5	5.3	8.7	11.0
1.8	5.5	8.9	10.6
2.1	6.2	9.1	10.4
2.3	6.3	9.3	10.7
2.5	6.3	9.5	11.4
2.7	7.1	9.8	11.8
2.9	8.9	10.1	12.2
3.1	9.7	10.3	12.9
3.2	10.4	10.6	14.4
3.3	12.3	11.0	13.3
3.4	17.2	11.3	13.0
3.5	16	11.6	13.1
3.6	18.2	12.3	13.6
3.7	15.3	12.6	14.3
3.8	16.8	13.0	13.5
3.9	16.3	13.3	13.4
4.0	17.3	13.8	14.1
4.1	16.1	14.3	14.3
4.2	14.8	15.3	14.7
4.3	13.9	15.8	15.9
4.4	12.8	16.3	15.8
4.5	12.8	16.8	16.2
4.6	11.9	17.3	15.2
4.7	11.6	17.8	16.0
4.8	10.8	18.3	16.4
4.9	10.0	19.3	17.2
5.0	9.7	20.3	17.5
5.1	8.7	22.0	17.4
5.2	8.4	25.0	17.1
5.3	8.5	28.0	17.5
5.4	8.6	30.0	18.3
5.5	8.6	32.0	18.0
5.7	8.3	35.0	17.3
5.9	8.2	40.0	17.1
6.0	8.8	45.0	17.0
6.1	9.1	50.0	16.1
6.2	9.1	55.0	15.7
6.3	8.7	60.0	15.6
6.5	8.7	65.0	15.2
6.8	9.1	70.0	14.3
7.1	9.7	80.0	13.5
7.3	10.1	90.0	13.0
7.5	10.5	100	12.8
7.7	11.2	120	11.5
7.9	11.7	150	10.6
8.1	10.8	200	9.3
8.3	11.3		

These experimental results are also plotted in Figure 1b, together with those of our previous measurements^54^ for higher electron energies (400–5000 eV), the measurements from Field et al.^55^ for lower energies (0.1–1 eV) and the values recommended by Itikawa.^30^ As seen in this latter figure, the present TCS data are consistent with the other two sets of experimental data for higher and lower energies, respectively. Since the estimated uncertainties of these experimental data are below 5%, we can conclude that the present results form a reliable ensemble of total electron scattering reference cross sections of CO_2_ for impact energies from 0.1 to 5000 eV. When compared with Itikawa’s data, we found, in general, good agreement. However, some discrepancies appearing at intermediate and low energies deserve a deeper discussion. Below 1 eV, Itikawa followed the recommendation of Zecca et al.^56^ of averaging the available data from Ferch et al.^57^ and Buckman et al.^58,59^ However, we should note that more recent measurements from Field et al.,^55^ with extremely good energy resolution (about 2 meV), also need to be considered. These more recent results show a considerable increase of the TCS for incident energies below 1 eV. This behavior is compatible with the existence of a near zero energy virtual state as confirmed by Lee et al.^40^

Accordingly, the e-CO_2_ TCS values that we recommend here are shown in Table 2. As mentioned above, these are based on the present measurements and those from refs (54 and 55) (see Figure 1b) and they clearly improve the accuracy and level of detail of those recommended by Itikawa^30^ in 2002. Consequently, we will use the present recommended data as our reference values for the comparative study between electron and positron scattering cross sections of CO_2_ described in Section III.

**Table 2:** Recommended Total Electron Scattering Cross Sections (TCS) from CO_2_ Molecules in SI Units (See Text for Details)

energy (eV)	TCS (10^–20^ m^2^)	energy (eV)	TCS (10^–20^ m^2^)
0.1	62.8	20	17.3
0.15	48.2	25	17.1
0.2	36.5	30	18.3
0.25	29.2	40	17.1
0.3	26.2	50	16.1
0.4	19.5	70	14.3
0.5	15.2	100	12.8
0.7	10.3	150	10.6
1	7.6	200	9.3
1.5	5.3	300	7.4
2	5.8	400	5.91
2.5	6.3	500	5.12
3	9.3	700	4.06
4	17.3	1000	3.16
5	9.7	1500	2.42
6	8.8	2000	1.92
7	9.4	3000	1.36
10	12	4000	1.07
15	14.5	5000	0.893

### II.B Dissociative Electron Attachment Measurements

From 1 to 40 eV, the above TCS measurements provide additional information, to the Itikawa’s recommended data, by showing structures in the TCS. These features (resonances) correspond to the formation of transient negative ions (electron attachment processes), which finally decay to the neutral molecular state or lead to different anionic/neutral fragments via dissociative processes (DEA processes). In the case of CO_2_, the most representative electron attachment processes, excluding the dissociation into neutral fragments, can be represented as
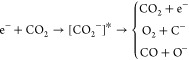
1

The aforementioned prominent resonance within 3.1–5.2 eV corresponds to a “shape resonance”.^60^ The shape of the potential well formed between the attractive Coulombic potential and the repulsive centrifugal barrier allows that incident electrons with specific energies are resonantly trapped by the target. Recommended data from ref (30) shows this resonance to occur in both the elastic and the total cross section. In elastic collisions, the total kinetic energy of the system projectile-target remains constant after the collision. Hence, strictly speaking, electron attachment processes cannot be considered as elastic collisions. However, as simple trapping mechanisms are determined by potential barriers, they commonly appear in the elastic scattering calculations. The present TCS measurements (Figure 1a) clearly show that this resonance has as a more pronounced maximum which is split into three peaks. This feature has been identified as a ^2^Π_u_ symmetry shape resonance,^61^ and the peak structure we found is similar to that observed by Dressler and Allan^62^ in the O^–^ formation yield by electron attachment to CO_2_. They attributed this structure to the final vibrational states of the formed CO molecule together with the vibrational structure of the intermediate CO_2_^–^ anion. These features were confirmed later by Cicman al.,^63^ and more recently by Fan et al.,^64^ assigning the main structures to the vibrational states of CO over much weaker and narrower structures due to the transient CO_2_^–^ anion. Above this π_u_ shape resonance, some structures can be distinguished in the present data which are not visible in the Itikawa’s recommended TCS values. For example, we found a broad structure from 5.2 to 5.9 eV. This feature was initially discussed by Chantrell et al.,^35^ who found it to peak at 5.77 eV, just above the threshold for excitation of the ^1^B_2_ electronic state of CO_2_, but after a detailed analysis of possible mechanisms leading to resonance formation they concluded that it maybe consists of an overlapping of various sharp resonances corresponding to relatively long lifetimes.^35^ We also found three structures peaked at 6.1, 7.9, and 8.5 eV, which correspond to the core-excited Feshbach resonances identified by Chantrell et al.^35^ Within these features is included the 8.2 eV resonance, which has been studied in detail by Slaughter et al.^37^ By combining the results of momentum imaging spectroscopy with *ab initio* theory, they proposed that it is initiated by the attachment of the electron to a ^2^Π_u_ doubly excited state that interacts with a lower ^2^Π shape resonance through a conical intersection and finally dissociates to electronic ground-state products. The next feature we found consisted of peaks within the range of 10.6 to 11.3 eV, which was also identified in ref (34). Spence and Schulz^32^ associated this resonance with the formation of the O_2_^–^ fragment. The next peak we observed, within 12.6–13.0 eV, was also attributed to the formation of O_2_^–^ in ref (31). Note that the shoulder we found at around 9.1–9.8 eV may indicate the presence of a new resonance, not identified at the moment. Other resonances distinguishable in the present TCS data at 12.6, 15.8, and 16.8 eV, as well as minor structures at 13.4 and 14.3 eV, are difficult to analyze due to the various excited states overlapping and the ionization continuum starting at 13.77 eV. The local maximum we found in the energy range of 17.0 to 22.0 eV may correspond to the C^–^ formation observed by Spence and Shultz.^32^ Note that around 30 eV and above 40 eV we can distinguish different shoulders, that already have been observed by Hoffman et al.^65^ and Smytkowski et al.,^66^ which can be again related to core-excited resonances.

To investigate the dissociative anion formation via electron attachment to CO_2_, we report new measurements of the relative O^–^ production yield over the energy range where we found the above resonances, i.e., from 3 to 52 eV. For this purpose, we utilized a momentum imaging spectrometer, which has been used in previous studies.^67−69^ Briefly, a stainless-steel capillary was employed to produce an effusive jet of CO_2_ molecules, which was crossed at 90° with a pulsed electron beam in a coaxial magnetic field. The absolute electron energy was determined and checked periodically by measuring ion yield across the thermodynamic threshold for O^–^ production from CO_2_, while the full 4π steradian ion collection of the momentum spectrometer was calibrated against the well-known O^–^ momentum distribution from DEA to O_2_. The time-of-flight and positions of each ion hit are recorded by a time- and position-sensitive detector in an event list.

**Figure 2:**
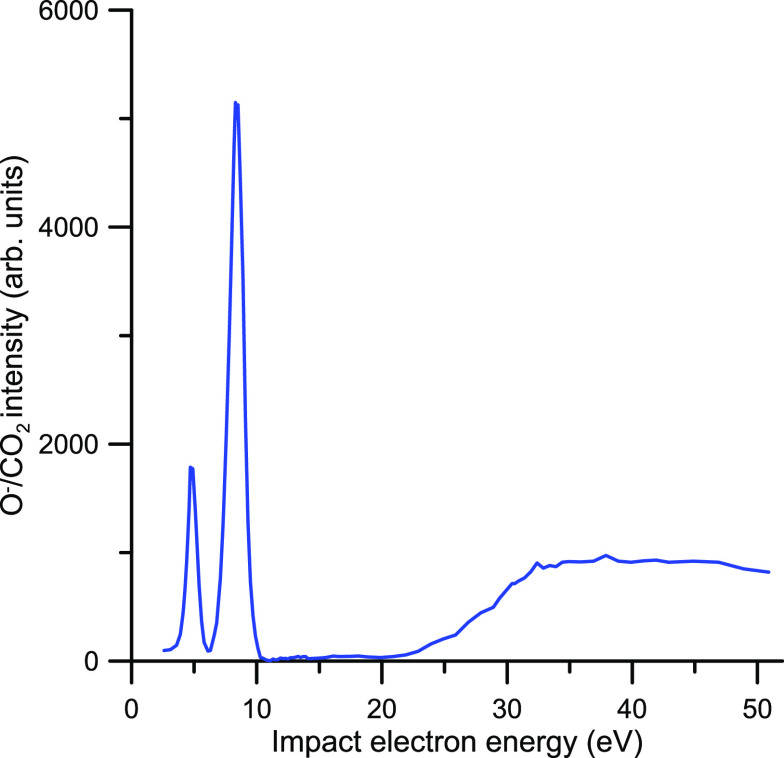
Relative O^–^ production yield by electron attachment to CO_2_.

Measurements have been performed by recording the O^–^ detected signal for electron incident energies ranging from 3 to 52 eV. The O^–^ signal is integrated over all the emission angles and kinetic energies for select time-of-flight and position windows on the detector, for efficient suppression of the scattered electron background. The corresponding results are plotted in Figure 2, showing that two prominent peaks centered at 4.7 and 8.3 eV dominate the O^–^ production by electron attachment to CO_2_ for the lower energies. The shape and positions of these two peaks agree with those shown by Orient and Srivastava.^67^ Although, according to the relative intensity of the resonances displayed in our TCS measurements, the main contribution to the electron attachment cross section corresponds to the resonance at around 4 eV (see Figure 1a), the O^–^/CO_2_ yield shown in Figure 2 indicates that the maximum anionic dissociation takes place at around 8.2 eV. This result may suggest that electron detachment could be the main relaxation mechanism via the low energy ^2^Π_u_ resonance. The theoretical model proposed by Vanroose et al.^41^ showed that vibrational bending of the CO_2_ molecule facilitates the connection between the aforementioned near to zero energy virtual state and the ^2^Π_u_ resonance producing a conical intersection of their respective potential curves. This model also explains the observed increase of the differential cross sections in the forward direction for the lower energies. Although the ground state of CO_2_ does not possess a permanent dipole, when the molecule is bent the induced dipole moment modifies the structure of the scattering cross sections. In addition, McCurdy et al.^42^ reported results of resonant vibrational excitation of CO_2_ by electron impact via the ^2^Π_u_ shape resonance. These evidence confirm that different relaxation ways of this resonance are competing with the DEA process, thus leading to a reduction of anion signal intensity. For higher energies, above 20 eV, the anion yield increases in magnitude to reach a “plateau” at about 40 eV. This smooth energy dependence of the O^–^ production yield suggests that nonresonant ion pair (anion and cation) processes are the dominant contribution to the anion fragment at such high energies. The local maxima at 30 and 37 eV, which we found in the TCS values, could indicate the presence of some resonant electron attachment processes at these energies although the present anion yield measurements are not able to confirm this point. Some weak maxima are visible on the O^–^ yield curve, but are within the uncertainty limits (10%) and so we are not able to confirm their existence. More sophisticated experiments, detecting anions and cations in coincidence to separate the contribution of the ion-pair production from a possible resonant anion dissociation would be required to elucidate this point.

## III Electron and Positron Scattering Cross Section Calculations

Our screening corrected additivity rule, based on the independent atom model (IAM-SCAR), for electron scattering from polyatomic molecules was described in 2004 (see ref (28) and references therein). Some years later, the effect of interferences in both the differential and integral cross section calculations was introduced to our calculation procedure (IAM-SCARI).^29^ For electron impact energies above 10 eV, this method has been proven to provide reliable differential and integral elastic, as well as integral inelastic and total scattering cross sections, for a wide variety of molecular targets (see ref (70) and references therein). This method was initially translated to the case of positron scattering from atoms,^10^ and then subsequently for molecules.^21^ Basically, it assumes that a molecule can be represented by an aggregate of independent atoms. The scattering potential for the constituent atoms, as a function of the scattering coordinate (*r*), can be represented by a complex expression given by
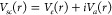
2where the real part (*V*_*e*_(*r*)) represents the elastic scattering and the imaginary part (*V*_*a*_(*r*)) represents the inelastic processes which are considered as absorptions from the incident beam. The elastic potential for electrons [*V*_*e*_(*r*)]_*e*_ contains three terms:
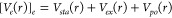
3

Namely, the static (*V*_*sta*_(*r*)) term represents the electrostatic interaction which is described at the Hartree–Fock level, the exchange potential (*V*_*ex*_(*r*)) accounts for the indistinguishability of both the incident and scattered electrons and the polarization potential (*V*_*po*_(*r*)) which introduces the distortion of the target electron cloud during the collision (see Blanco and García^70^ for details on the formulation of these potentials).

Similarly, in the case of positrons the real part of the potential representing the elastic scattering can be written as
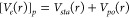
4where the main difference with that for electrons consists of the obvious absence of the exchange term thus giving more relevance to the polarization term. For the *V*_*po*_(*r*) polarization potentials we used a modified version of those proposed by Jain^71^ and O’Connel and Lane,^72^ for the scattering of positrons and electrons, respectively, by atoms. In both cases, they can be represented by
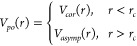
5where *V*_*cor*_(*r*) accounts for the correlation energy corresponding to an electron^72^ or positron^71^ entering the target electron cloud (considered as a Fermi gas), and therefore, it depends on the electron density, while *V*_*asymp*_(*r*) = −α_*d*_/2*r*^4^ – α_*q*_/2*r*^6^ represents the asymptotic behavior of *V*_*po*_(*r*) as a function of the dipole (α_*d*_) and quadrupole (α_*q*_) atomic polarizabilities of the target and *r*_*c*_ is the crossing point of both the *V*_*cor*_(*r*) and *V*_*asymp*_(*r*) functions.

Note that the above polarization terms are similar for electrons and positrons and are repulsive in both cases. However, the static term is repulsive for electrons, while for positrons it is attractive. As a result of this fact, the global contribution of these terms to the elastic potential is higher for electrons than for positrons even for energies high enough to neglect the exchange term. This is a key point of the present comparative study and will be discussed later when comparing the available results. In both cases, the above complex potential (eq 2) allows a partial wave expansion of the scattering equation leading to the calculation of the corresponding complex phase-shifts. These are related to the differential elastic cross sections (DCS), which by integration over the entire scattering angular range provides the integral elastic cross sections (ICS) and finally, by applying the optical theorem, the total scattering cross sections (TCS). Although the elastic scattering is represented by the real part of the above potential (*V*_*e*_(*r*)), the calculation procedure is also sensitive to its imaginary part (*V*_*a*_(*r*)); thus, in order to obtain reliable elastic cross section values, the absorption potential needs to be properly defined.

With respect to the absorption potential (*V*_*a*_(*r*)), representing the inelastic scattering, in the case of electrons, we used our nonempirical improved formulation of the model potential initially proposed by Staszewska et al.^73^ These improvements include restoring the local velocity during the collision, allowing for electron screening effects, and accounting for relativistic and many-body corrections.^74^ For positrons, we adopted the absorption model potential proposed by Reid and Wadehra.^75,76^ Note that the critical point in using this kind of potential is the accurate definition of the threshold excitation energy (Δ). By definition, this threshold is coincident with the excitation energy of the lowest excited state of the atom. In these conditions, the absorption potential provides integrated values of the inelastic cross sections as a whole, without distinction between the different inelastic channels. However, as shown in previous studies,^77^ by alternatively using the ionization energy limit as threshold energy (Δ_ion_), we can extract the total ionization cross section from the integral inelastic cross sections. Similarly, in the case of positrons,^78^ using the positronium formation limit as the threshold energy (Δ_p_), we can separate the positronium formation cross sections from the total ionization cross sections. In this case, as positronium formation typically only occurs over a quite limited impact energy range, an energy-dependent Δ_p_ has been adopted.^78^ As mentioned above, our IAM-SCARI procedure has been used to calculate the electron and positron scattering cross sections from CO_2_ through the calculated differential and integral cross sections for the C and O atoms.

## IV Discussion on Electron and Positron Scattering Cross Section Data

**Figure 3:**
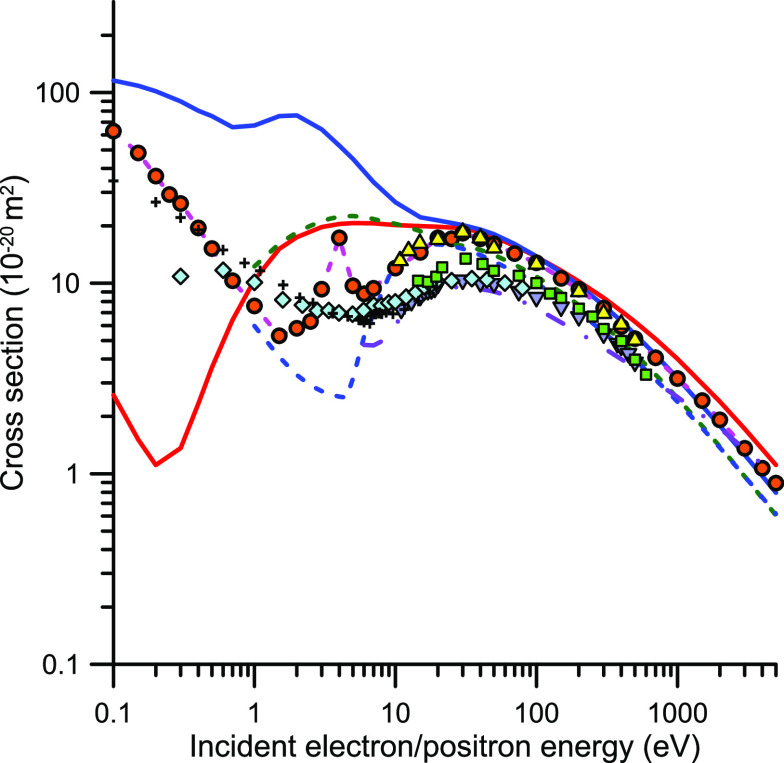
Total electron and positron scattering cross section (TCS): orange −, present e-TCS calculation; blue −, present p-TCS calculation; orange ●, present recommended e-TCS; green ---, e-TCS calculated by Billah et al.;^80^ blue ---, p-TCS calculated by Billah et al.;^80^ violet -·-, p-TCS calculated by Shing et al.;^81^ yellow ▲, experimental e-TCS from Kwan et al.;^79^ blue ▼, experimental p-TCS Kwan et al.;^79^ light blue ◆; experimental p-TCS from Sueoka et al.;^82,83^ green ■, experimental p-TCS from Charlton et al.;^84^ +, experimental p-TCS from Zecca et al.^85^

In order to discuss the accuracy that we can assign to these calculations, electron and positron scattering cross sections have been compared with the corresponding data available in the literature, having in mind that our reference data are the recommended TCS values shown in Table 2. The present calculated total scattering cross sections for electrons (e-TCS) and positrons (p-TCS), from 0.1 to 5000 eV, together with our recommended data for electrons are plotted in Figure 3. Representative experimental and theoretical TCS values available in the literature^79−85^ are also included in this figure for comparison.

As shown in Figure 3, there is a good level of agreement, within 10%, between the calculated and recommended TCS values for electrons at impact energies of 20 eV and above. Below 20 eV, our calculated data tend to be lower in magnitude than the experimental ones due to the poor description of the scattering process given by IAM-SCAR method at such low energies, where the molecular properties are obviously relevant. We consequently will exclude, in our further discussions, our calculated cross section data below 20 eV. Nonetheless, it is interesting to note that, for such low energies, using the same level of approximation for electrons and positrons, the polarization potential is much more relevant for positrons than for electrons, producing an intensification of the p-TCS magnitudes of several order of magnitude with respect to those for the electrons. Note that from 20 to 100 eV our calculated TCS for electrons and positrons are coincident. However, for higher impact energies, the TCSs for positrons tend to be lower in value than those for electrons, reaching a maximum discrepancy of about 30% at 5000 eV. This is in contradiction with the generally observed tendency toward a merging of the electron and positron cross section curves at the highest energies.^79^ Recently, a model potential calculation of electron and positron scattering cross sections has been published by Billah et al.^80^ The theoretical method used in that study is similar to that of the present calculations, but instead using the relativistic Dirac equation, so comparison between both sets of results can be relevant to this discussion. Thus, TCS results of ref (80), for electron and positrons within the (1–5000 eV) impact energy range, are also plotted in Figure 3. The first feature of this calculation that we can observe from this figure is that, for impact energies above 20 eV, the TCS results for positrons and electrons are coincident to within 10%. This is a very surprising result, in clear contradiction with the experimental data, that will deserve further investigation. In addition, the TCS results for electron scattering from CO_2_, calculated by Billah et al.,^80^ are lower in magnitude than the present experimental reference data by about 48% at 5000 eV. With respect to the TCS for positrons, results from ref (80) are in reasonable agreement (within 10%) with the present calculation for impact energies above 20 eV but tend to be much lower in value below this energy. Among other previous calculations, cited in ref (25), relevant to this study include positron data from Singh et al.,^81^ which are calculated with a spherical complex optical potential method. As may be seen in Figure 3, results using this latter formalism, for energies below 400 eV, are remarkably lower in magnitude than the above calculations. Since the mentioned disagreement, using similar or different theoretical approaches, mainly occurs between 20 and 400 eV, we can incorporate into the discussion the experimental TCS data from Kauppila’s group^65,79^ and those from Sueoka’s group,^2,82,83^ where in both cases the same experimental apparatus has been used for electrons and positrons. Both experimental sets of data confirm that in the (20–400 eV) energy range the positron scattering TCSs for CO_2_ are lower in magnitude that those for electrons. By including the results from Charlton et al.^84^ and Zecca et al.,^85^ we can observe that the experimental TCS data for positrons, in this energy range, show a general agreement between them, and they agree better with the calculation of Singh et al.^81^ than with the present one and that from Billah et al.^80^

**Figure 4:**
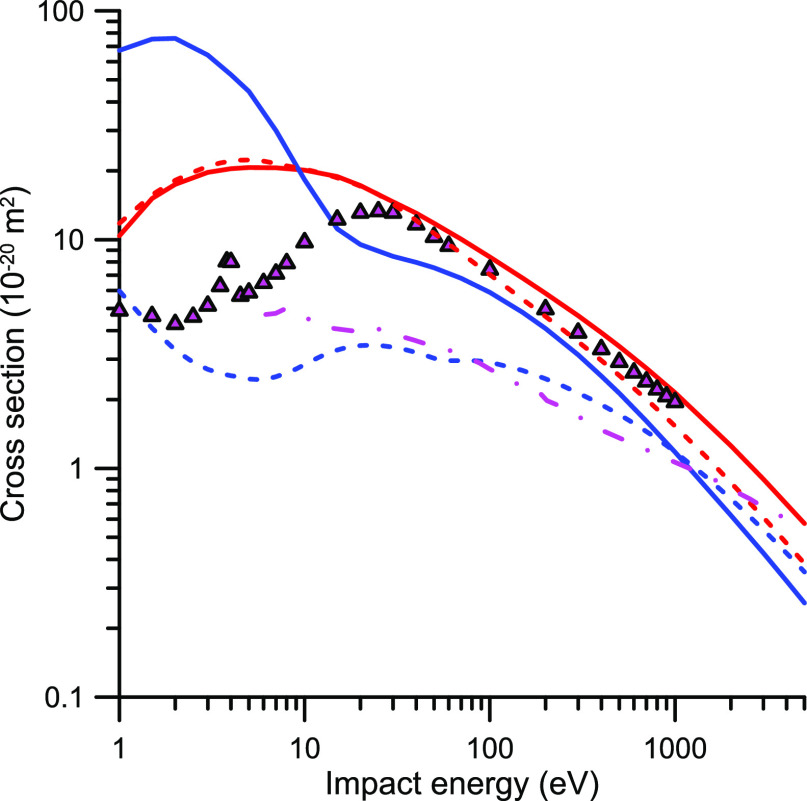
Integral elastic cross sections (IECS) for electron and positron scattering by CO_2_. red −, present e-IECS calculation, blue −, present p-IECS calculation; red ---, e-IECS calculated by Billah et al.;^80^ blue ---, p-IECS calculated by Billah et al.;^80^ -·-, p-IECS from Singh et al.^81^ calculation; pink ▲, e-IECS recommended by Itikawa.^30^

In order to understand the origin of these discrepancies, we have analyzed the contribution of the main scattering channels to the above TCS values. Our calculated elastic and inelastic integral cross sections for electron and positron scattering from CO_2_ are plotted in Figure 4.

### IV.A Elastic Scattering Cross Sections

The elastic scattering cross sections are displayed in Figure 4. As shown in this figure, for energies above 20 eV (where the present IAM-SCARI calculation is expected to be reliable to within 10%), the integral elastic cross sections for positrons are always lower than that for electrons. This can be justified, at least in part, by the absence of the exchange term in the scattering potential for positrons (see eqs 2 and 3). However, the exchange potential for electrons vanishes with increasing energies, while the aforementioned difference persists up to 5000 eV. This seems to indicate that essential differences between the electron and positron scattering potentials act against the merging of their respective cross sections for increasing energies, at least up to 5000 eV. For the potential we used in this calculation, this essential difference can be explained by the aforementioned different signs of the terms included in eqs 2 and 3. While the polarization potential term leads to a repulsive force in both cases, the static potential generates repulsive and attractive forces in the case of electrons and positrons, respectively. The attractive force of the latter partially compensates for the repulsive polarization force, leading to calculated cross section values lower than those of the former. However, this essential difference is not apparent in the calculation of Bilhah et al.^80^ where the integral elastic cross sections for positrons, being lower than those for electrons, tend to merge as the energy increases. Nonetheless, both calculations are in agreement with the Born approximation in the sense that, for very high impact energies, the energy dependence of the cross section tends to its predicted *E*^–1^ behavior. However, the calculations of Singh et al.^81^ show a much flatter energy dependence, around *E*^–0.4^, for energies above 3000 eV. Note that this energy dependence, in order to give the appropriate asymptotic behavior, would need a sudden increment of the negative slope for energies above 5000 eV which would be really difficult to explain from a physical point of view. Unfortunately, no experimental data are available to compare with the calculated energy dependencies of the IECSs. Thus, the discussion remains open to further evidence, but at this point we can conclude that most of the discrepancies found between the calculated p-TCS for energies ranging from 20 to 400 eV are due to an overestimation of the present IAM-SCAR data.

### IV.B Inelastic Scattering Cross Sections

**Figure 5:**
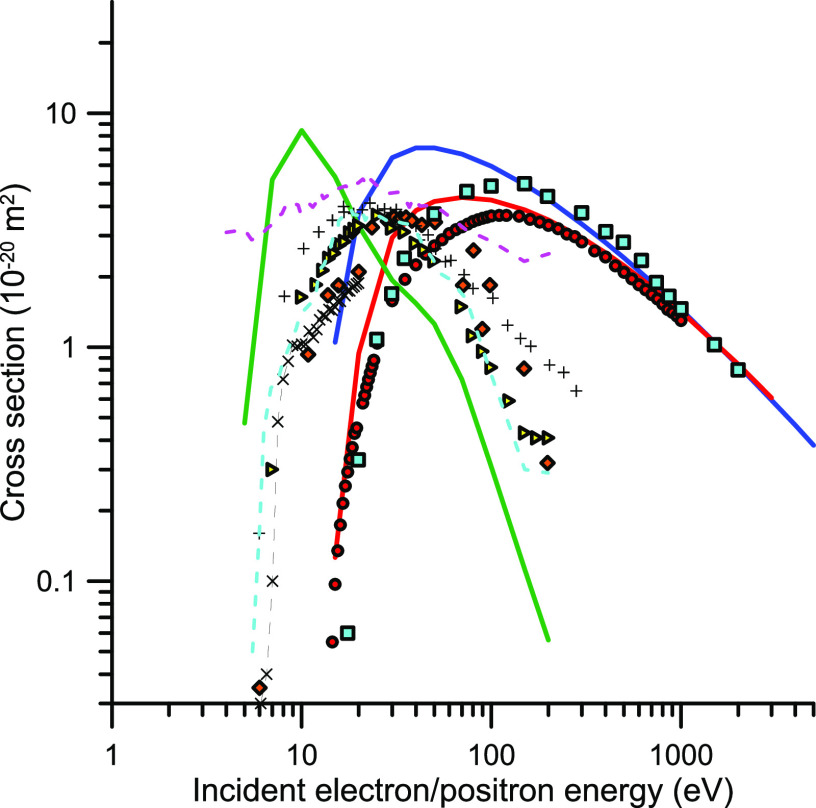
Ionization cross sections (ION) of CO_2_ by electron and positron impact: red −, present e-ION calculation; blue −, present p-ION calculation; red ●, e-ION recommended by Itikawa;^30^ light blue ■, experimental p-ION from Bluhme;^92^ ×, experimental total ionization (p-ION + positronium formation) cross section from Laricchia and Moxom;^93^ light blue −, present positronium formation cross section calculation; orange ◆, experimental positronium formation from Bluhme,^92^ +, Cooke et al.^94^ experimental positronium formation cross section; yellow ●, Murtagh et al.^95^ experimental positronium formation cross section; upper (pink ---) and lower (light blue ---) limits of the positronium formation cross sections given by Kwan et al.^96^

Electron and positron impact ionization cross sections are plotted together in Figure 5. With respect to electron collisions, our present calculation shows excellent agreement with the data recommended by Itikawa^30^ for energies above 100 eV. From 20 to 100 eV the present calculation overestimates those recommended in ref (30) by about 30%, mainly due to the limitations of the present single atom representation around the ionization threshold. The recommended values by Itikawa^30^ are based on accurate experimental data (see ref (30) for details), however we should note that they perfectly agree, within 7%, with the BEB^86^ calculation of Hwang et al.,^87^ which is clearly supporting these recommended data. In the case of positrons, the situation is more complicated. Effective ionization by positron impact can be achieved either by positronium formation or direct ionization processes. Representative positronium formation and direct ionization cross sections are shown in Figure 5. For both ionizing processes, our calculation is not reliable around their respective thresholds, but as demonstrated in previous publications,^77,88^ it gives a good indication as to the maximum cross section values and their respective asymptotic behavior for increasing energies. As shown in Figure 5, our respective ionization cross section calculations for positrons and electrons merge for impact energies above 200 eV, thus confirming the predictions of the first Born approximation. A similar behavior was found by previous calculations from Tóth et al.,^89^ Campenau et al.^90^ and Singh and Antony^91^ (for simplicity these calculations are not plotted in Figure 5 but comparison between them can be found in ref (91)). If we compare with the experimental data, the direct ionization cross section measurements from Bluhme et al.,^92^ beyond the maximum cross section value, show good agreement with the present calculation. From the ionization threshold (about 13 eV) up to the maximum cross section value (about 100 eV), as expected, our calculation does not reproduce the observed energy dependence of the ionization cross section.

Concerning the positronium formation cross section, again our calculation just gives an indication of its magnitude beyond the maximum cross section value, at about 20 eV. However, by comparing with the available experimental data,^93−96^ we can estimate reasonable values of the positronium formation cross section over the whole energy range. As can be seen in Figure 5, this energy range extends from about 7 up to 300 eV.

**Figure 6:**
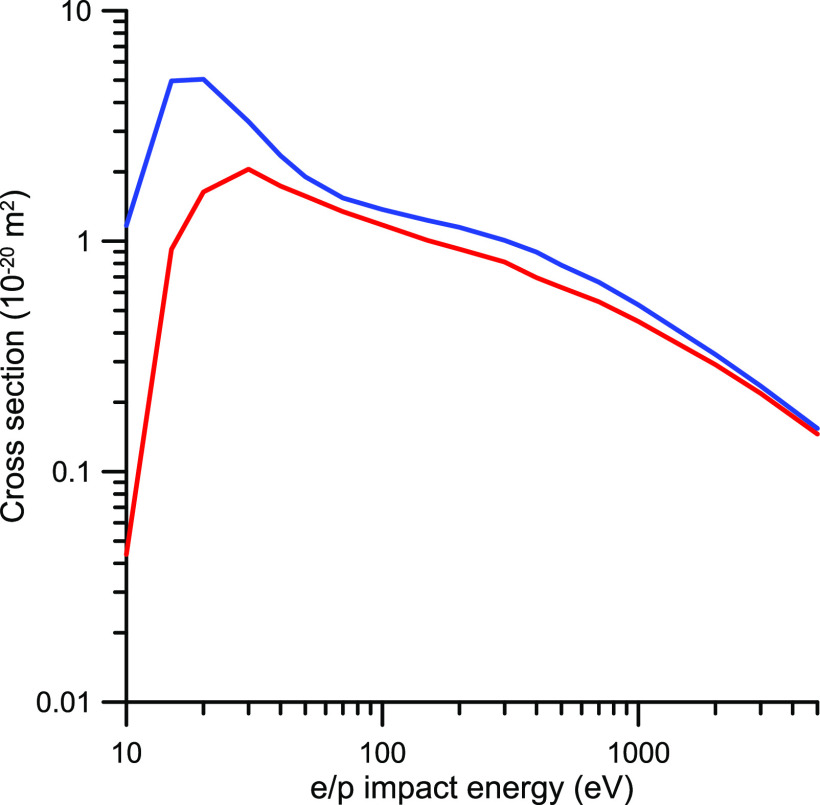
Electronic excitation cross sections of CO_2_ by electron (red −) and positron (blue −) impact.

With respect to electronic excitation, both for electron and positron scattering cross sections, our calculation provides integral data given by the difference between the TCS and the ICS corresponding to the aforementioned channels, i.e., elastic, ionization, and positronium formation. Results derived with this procedure are shown in Figure 6. Note that Itikawa’s compilation does not provide the total electron electronic excitation cross section but provides it just for the excitation of a few states at the given impact energies.

As shown in Figure 6, our calculated excitation cross sections for positron impact are higher in magnitude than the corresponding cross sections for electrons, but they tend to merge for increasing energies, as predicted by the Born approximation. Whether excitation of molecules by positrons should be more or less efficient than by electrons has been discussed in previous publications for different atomic and molecular targets.^27^ Although these studies suggest that, due to the absence of the exchange potential, positrons have less probability than electrons to excite states requiring to change the spin, and this may lead to a lower excitation cross sections for positrons, this does not happen in the case of CO_2_. Probably, in this case, the relatively large attractive electron cloud of the molecule and its screening effect on the repulsive charge of the nuclei facilitate the collision of the incoming positrons with the target electrons.

## V Recommended Electron and Positron Scattering Cross Section Data

Concerning electron scattering by CO_2_, the consistency of the data recommended by Itikawa^30^ can be checked by adding his elastic, ionization, vibrational excitation, and electron attachment cross sections to the present excitation cross sections and comparing the results with our recommended TCS data listed in Table 2. By following this procedure, we found that both quantities agree within 3–15% for the whole energy range considered here (0.1–5000 eV). This is a good result, if we consider that integral cross sections for the above-mentioned scattering channels have typically uncertainties of about 10–20%. We can then conclude that, concerning electron scattering, Itikawa’s recommended data are still operative. However, as a note of caution, the electron attachment is not fully treated as an independent process by Itikawa.^30^ It is partially accounted for from the observed anion fragmentation and part of the prominent shape resonance around 3.6–3.8 eV is included in the recommended elastic cross section (see Figure 3). In order to derive a complete electron attachment cross section data set, we proceed in a similar way as that we followed in previous studies.^97,98^ Since electron attachment processes are depicted in our TCS measurements as local maxima, we can evaluate their respective contributions to the TCS from a simple analysis of the cross section curve as a function of the impact energy. For each impact energy, the amount of the cross section to be assigned to the resonant process is the result of subtracting from the total cross section those of the corresponding nonresonant channels at that energy, i.e. the elastic scattering for the shape resonance and the elastic plus vibrational and electronic excitation channels for the Feshbach and core excited resonances (see the analysis of these resonances in Section II).

As already mentioned, the electronic excitation cross sections are not available in Itikawa’s compilation, but we here recommend a set of data which is consistent with the present TCS shown in Table 2 and the other scattering channels (elastic, attachment, vibrational excitation and ionization) discussed above. This set of self-consistent data is shown in Table 3.

**Table 3:** Self-Consistent Set of Electron Scattering by CO_2_ Cross Section Data (in 10^–20^ m^2^ Units,) Based on the Recommended Data from Reference (30) and the Present e-TCS Shown in Table 2

*E* (eV)	elastic	vibrational excitation	electronic excitation	electron attachment	ionization
0.1	62.6	0.2			
0.15	47.9	0.25			
0.2	34.8	1.70			
0.3	24.4	1.80			
0.4	16.7	2.76			
0.5	12.9	2.30			
0.7	8.63	1.67			
1	6.30	1.30			
1.5	4.73	1.29			
2	4.37	1.23	0.2		
3	5.02	1.43	0.556	2.30	
4	5.56	1.49	0.600	9.65	
5	6.00	0.879	1.41	1.41	
7	7.25	0.502	1.18	0.462	
10	9.95	0.247	0.912	0.891	
15	12.5	0.283	1.62	0.001	0.097
20	13.4	0.191	2.21	1.01	0.491
30	13.4	0.171	2.61	0.568	1.58
40	11.9	0.200	2.75		2.25
50	10.5	0.180	2.71		2.71
70	8.95	0.150	1.93		3.27
100	7.55	0.130	1.48		3.64
150	5.98		1.05		3.57
200	5.07		0.91		3.32
300	4.01		0.57		2.82
400	3.39		0.09		2.43
500	2.98		0.05		2.09
700	2.45				1.68
1000	1.99				1.30
2000	1.17				0.774
3000	0.833				0.556
4000	0.648				0.435
5000	0.534				0.359

According to the procedure for electrons, to derive a complete and consistent data set for positron we should start by proposing a reliable TCS reference data set. From the discussion in Section III, we have no special reasons to decide which TCS experimental results could be more accurate than the others. Although using transmission-beam techniques provides accurate TCS values, at least in terms of statistical uncertainties, it is well-known that these techniques may be affected by systematic errors associated with the existence of pressure gradients, the geometry of the interaction region and the acceptance angle of the detector. These error sources affect in a different way according to the different available techniques.^99^ After a careful analysis of the accuracy of the experimental data available in the literature, Brunger et al.^7^ recommended a set of p-TCS cross sections for CO_2_ with uncertainty limits of less than 10%. These uncertainties are taken from the original publications, and do not include corrections connected with acceptance angle limitations.^99^ These limitations tend to lower the measured cross section values so we can expect that the true total cross section could be systematically higher than the recommended values. In order to minimize the effect of possible systematic errors in the experimental data, accounted for in the Brunger et al.^7^ compilation, we have renormalized these recommended data to the average value derived from the experimental TCS at 20 eV available in the literature. This average value was 6.25% higher than that recommended in ref (7), and thus the present recommended TCS values (see Table 4) are the latter multiplied by 1.0625 with a random uncertainty of about 7%. Our TCS calculation, renormalized at 500 eV, has been used to extrapolate the experimental values up to 5000 eV. The corresponding results are shown in Table 4.

**Table 4:** Recommended Positron Scattering from CO_2_ Cross Sections in 10^–20^ m^2^ Units

*E* (eV)	elastic	vibrational excitation	electronic excitation	positronium formation	direct ionization	total cross section
0.1	36.5					36.5
0.15	31.4					31.4
0.2	28.3					28.3
0.3	23.5					23.5
0.4	20.0	0.2				20.2
0.5	16.9	0.71				17.7
0.7	14.0	0.78				14.8
1	12.1	0.66				12.8
1.5	10.2	0.53				10.7
2	8.71	0.46				9.17
3	7.58	0.38				7.96
4	6.976	0.33				7.30
5	6.68	0.29				6.97
7	6.82	0.25		0.15		7.22
10	4.90	0.21	0.873	1.43		7.42
15	3.37		2.71	2.62		8.71
20	2.65		3.77	3.39	0.395	10.2
30	2.46		2.47 9	3.49	2.42	10.8
40	2.28		1.766	3.1	3.28	10.4
50	2.19		1.42 3	2.77	3.92	10.3
70	2.13		1.35	1.85	4.97	10.3
100	1.97		1.03	1.16	5.19	9.35
150	1.92		0.924	0.62	4.39	7.86
200	1.45		0.858	0.47	4.02	6.80
300	1.39		0.754	0.25	3.13	5.52
400	1.38		0.670	0.22	2.41	4.67
500	1.34		0.588		2.11	4.04
700	1.07		0.496		1.58	3.14
1000	0.908		0.396		1.07	2.37
2000	0.564		0.241		0.525	1.33
3000	0.353		0.176		0.410	0.939
4000	0.258		0.140		0.330	0.728
5000	0.199		0.115		0.280	0.594

**Figure 7:**
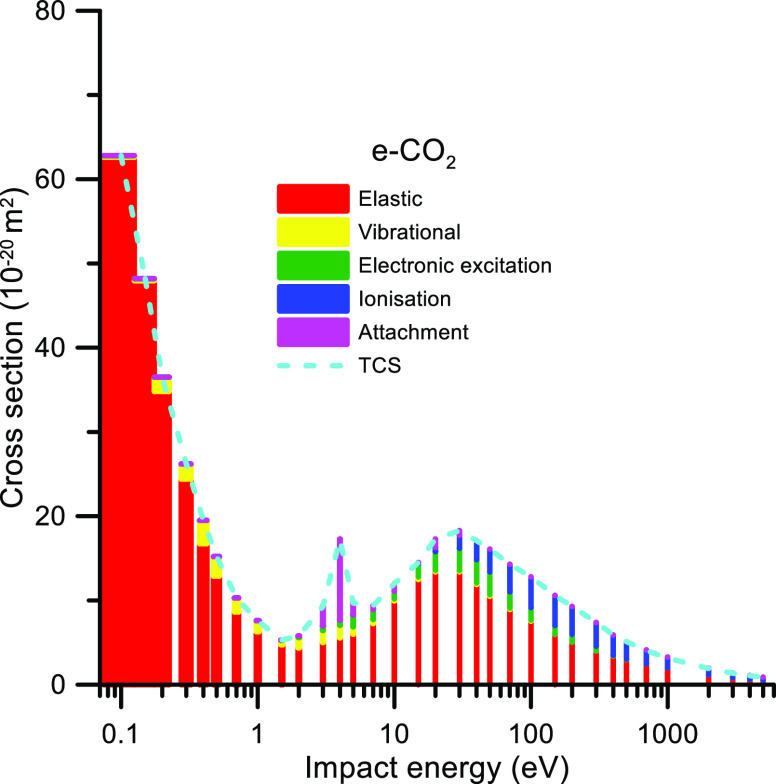
Contribution of each scattering channel to the CO_2_ total cross section for incident electron energies ranging from 0.1 to 5000 eV.

**Figure 8:**
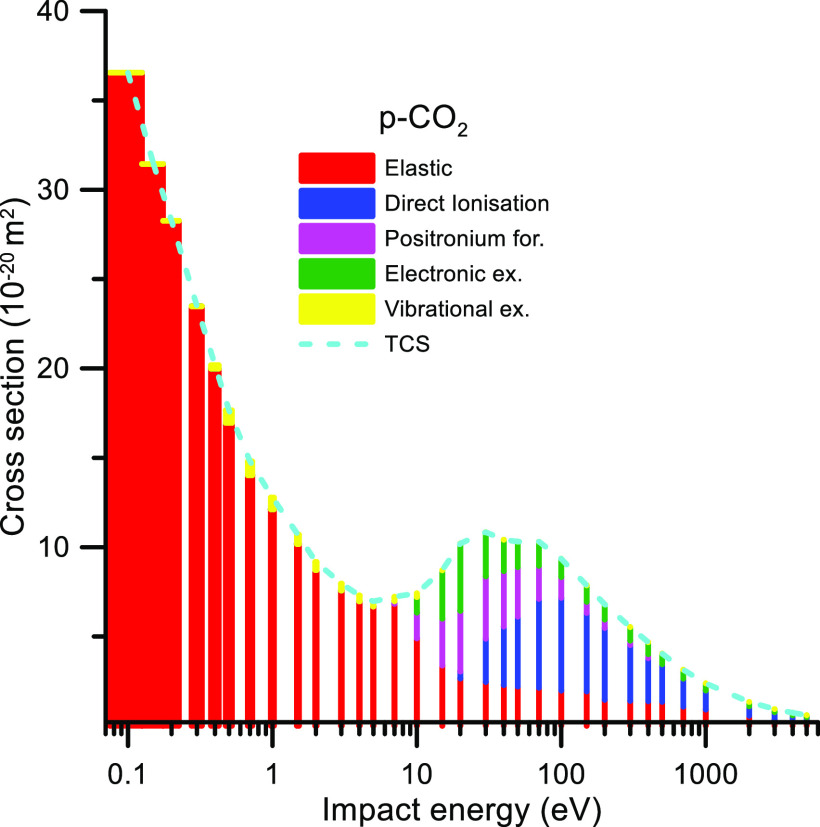
Contribution of each scattering channel to the CO_2_ total cross section for incident positron energies ranging from 0.1 to 5000 eV.

In order to illustrate the contribution of each scattering channel to the total cross section for electron and positrons, the corresponding elastic and inelastic scattering cross sections are plotted in Figures 7 and 8, respectively.

## VI Summary

The electron scattering cross sections from CO_2_, recommended by Itikawa,^30^ have been revisited and updated. By using a “State-of-the-Art” magnetically confined electron transmission apparatus, absolute total electron scattering cross sections have been accurately measured (within 5%) with an energy resolution of about 100 meV. These conditions allowed for the identification of some features in the e-TCS values, which were not shown in previous data compilations, and they have been identified as electron attachment resonances. The deconvolution of these resonances, from the TCS energy dependence curve, permitted the evaluation of the electron attachment to CO_2_ cross sections. In addition, the O^–^ production, via dissociative electron attachment, has been analyzed with a crossed beam apparatus provided with a momentum imaging spectrometer. This analysis confirmed previous measurements of the O^–^/CO_2_ production yield by electron attachment at 4 and 8.2 eV. However, we have demonstrated that O^–^ is also formed, through a broad continuum at higher energies (above 20 eV), which has been attributed to ion-pair formation processes. Although some weak structures at 30 and 37 eV appear over the continuum, the uncertainty limits of the present ion yield measurements do not allow for the definitive confirmation of the existence of dissociative electron attachment resonances above 30 eV. The consistency of Itikawa’s recommended data, complemented with the present electron attachment and electronic excitation cross sections, has been demonstrated by comparing the sum of all the considered scattering channels with our TCS reference data set for impact energies from 0.1 to 5000 eV.

It would be remiss of us not to mention the recent work on cross section data sets evaluation, using artificial intelligence (AI) and machine learning (ML) processes, that are being explored by the James Cook University group. This is mainly for atomic and molecular gases,^100−103^ but an extension to liquids has recently been examined.^104^ In the case of e-CO_2_ scattering in the gas phase, there seem to be enough cross section data that such AI/AL approaches might be gainfully applied, complementing the extensive recent work of Guerra, Alves and co-workers on this gas (see the review^105^), as well as other electron transport simulation procedures as such recently applied by Gracía-Abenza et al.^106^ to water vapor.

A similar procedure has been followed for positron scattering. In this case, the TCS reference data set has been based on the recommended data from Brunger et al.,^7^ and the cross sections of the different scattering channels have been derived by critically including previous results available in the literature complemented with our intermediate-high impact energy calculation.

Comparison between the present scattering cross sections, for electrons and positrons in the considered energy range, reveals that for the lower energies positron scattering is clearly dominated by polarization effects leading to a higher magnitude of the TCS than that corresponding to electrons (for impact energies below 10 eV). At intermediate energies, although the elastic scattering cross sections tend to be lower for positrons than for electrons (no exchange potential and opposite signs of the static and polarization potentials in the case of positrons), due to the increase of the inelastic cross sections for positrons (positronium formation and electronic excitation) the TCS for electrons and positrons tend to be similar for impact energies of 20 to 80 eV. At higher energies, from 100 to 5000 eV, the opposite sign of the polarization and static potentials for positrons still affects the TCS, always giving lower values for positrons than for electrons. This result does not agree with the convergence of the TCS values for positrons and electrons predicted by the first-Born approximation. However, we have shown that this convergence exists for the integral inelastic cross sections, while for the elastic scattering, due to the previously mentioned different polarization–static potential contributions, the cross section for positrons remains lower than that for electrons even at 5000 eV impact energy.

In spite of the great theoretical and experimental effort paid in the last 20 years to understand the electron and positron scattering processes for CO_2_, there are still open questions. Some of them have been discussed here, but the accurate description of the polarization effects at low impact energies, the evaluation of the true magnitude of the low-energy electron scattering integral cross section, the accurate inclusion of the positronium formation channel and the confirmation of the comparative magnitude of the electron–positron electronic excitation cross sections would require further consideration. We hope these challenges can motivate future theoretical and experimental studies on this subject.
